# Efficacy of Digital Health Tools for a Pediatric Patient Registry: Semistructured Interviews and Interface Usability Testing With Parents and Clinicians

**DOI:** 10.2196/29889

**Published:** 2022-01-17

**Authors:** Sarah Doyle, Rebecca Pavlos, Samantha J Carlson, Katherine Barton, Mejbah Bhuiyan, Bernadett Boeing, Meredith L Borland, Steven Hoober, Christopher C Blyth

**Affiliations:** 1 Emergency Department Perth Children's Hospital Perth Australia; 2 Wesfarmers Centre of Vaccines and Infectious Diseases Telethon Kids Institute Perth Australia; 3 University of Western Australia Perth Australia; 4 4ourth Mobile Mission, KS United States; 5 Perth Children's Hospital Perth Australia

**Keywords:** usability testing, REDCap, discharge instructions, acute respiratory infection, digital health technology, mobile technology, semistructured interview, pediatric acute respiratory infection, mobile phone

## Abstract

**Background:**

Acute respiratory infection (ARI) in childhood is common, but more knowledge on the burden and natural history of ARI in the community is required. A better understanding of ARI risk factors, treatment, and outcomes will help support parents to manage their sick child at home. Digital health tools are becoming more widely adopted in clinical care and research and may assist in understanding and managing common pediatric diseases, including ARI, in hospitals and in the community. We integrated 2 digital tools—a web-based discharge communication system and the REDCap (Research Electronic Data Capture) platform—into the Pragmatic Adaptive Trial for Acute Respiratory Infection in Children to enhance parent and physician engagement around ARI discharge communication and our patient registry.

**Objective:**

The objective of this study is to determine the efficacy and usability of digital tools integrated into a pediatric patient registry for ARI.

**Methods:**

Semistructured interviews and software interface usability testing were conducted with 11 parents and 8 emergency department physicians working at a tertiary pediatric hospital and research center in Perth, Western Australia, in 2019. Questions focused on experiences of discharge communication and clinical trial engagement. Responses were analyzed using the qualitative Framework Method. Participants were directly observed using digital interfaces as they attempted predetermined tasks that were then classified as *success*, *failure*, *software failure*, or *not observed*. Participants rated the interfaces using the System Usability Scale (SUS).

**Results:**

Most parents (9/11, 82%) indicated that they usually received verbal discharge advice, with some (5/11, 45%) recalling receiving preprinted resources from their physician. Most (8/11, 73%) would also like to receive discharge advice electronically. Most of the physicians (7/8, 88%) described their usual practice as verbal discharge instructions, with some (3/8, 38%) reporting time pressures associated with providing discharge instructions. The digital technology option was preferred for engaging in research by most parents (8/11, 73%). For the discharge communication digital tool, parents gave a mean SUS score of 94/100 (SD 4.3; A grade) for the mobile interface and physicians gave a mean usability score of 93/100 (SD 4.7; A grade) for the desktop interface. For the research data management tool (REDCap), parents gave a mean usability score of 78/100 (SD 11.0; C grade) for the mobile interface.

**Conclusions:**

Semistructured interviews allowed us to better understand parent and physician experiences of discharge communication and clinical research engagement. Software interface usability testing methods and use of the SUS helped us gauge the efficacy of our digital tools with both parent and physician users. This study demonstrates the feasibility of combining qualitative research methods with software industry interface usability testing methods to help determine the efficacy of digital tools in a pediatric clinical research setting.

## Introduction

### Acute Respiratory Infection in Children

Globally, acute respiratory infection (ARI) is a major cause of childhood morbidity and mortality; pneumonia alone is estimated to cause approximately 15% of all global deaths in children aged <5 years [1]. In Australia, children are known to have an average of 13 discrete episodes of ARI before the age of 2 years [2], and 1 in 4 presentations to Western Australian pediatric emergency departments (EDs) is due to ARI [3].

Our understanding of the burden of ARI in Australia is largely derived from hospital inpatient data [3]. Although the severe spectrum of ARI is important, most pediatric patients with ARI are discharged from the ED and recover at home or are treated by family physicians in the community. A better understanding of the burden and natural history of ARI in the community is required to improve our approaches to support parents and carers managing their sick child at home and to assess the efficacy of treatments.

### Digital Health Tools

Digital health is a broad term encompassing “digital information, data, and communication technologies to collect, share, and analyze health information for purposes of improving patient health and health care delivery” [4]. With smartphones now the new *normal computer* [5], there is increasing interest in how digital tools can be used to assist in improving clinical care and clinical research [6-8]. A recent systematic review of discharge communication practices in the pediatric ED examined 23 studies primarily focused on an education intervention involving “delivering information about an illness or instructions for managing care at home.” Of the 23 studies, 10 used technology to deliver this education intervention to parents, with the authors concluding that “technology-enabled *education* type interventions for parents had a positive impact on parent knowledge acquisition and adherence to guidelines, but were not effective in reducing unnecessary return visits to the ED” [9].

Research, particularly communicating study information and consent processes, may also be improved with digital tools [10]. Traditional paper-based informed consent involves a participant reading over long and complex text documents [11]. Evidence suggests that parents may have a poor understanding of the study information provided even when they have no other barriers to understanding (eg, limited English proficiency) [12]. The aim of integrating technology into research processes should not simply be to transform paper-based resources into digital resources; rather, technology may offer an opportunity to rethink and optimize existing processes. For example, a more participative electronic consent process can feature mobile technology with multimedia components such as video to better align with basic principles of human learning [13].

The ongoing COVID-19 pandemic has led to renewed interest in overcoming the challenges associated with the adoption of digital health tools. Strict data-protection and privacy regulations, a lack of funding, and complexity around interoperability of systems have been long-term challenges [14]. In addition, a lack of user-centered design of digital health tools is common. Unlike other industries such as aviation, in health care “the culture is still to train people to adapt to poorly designed technology, rather than to design technology to fit people’s characteristics” [15]. This was demonstrated in a large US cross-sectional survey where 870 physician users gave an average of an *F* grade (representing 0-60/100 on the System Usability Scale [SUS]) for the usability of their electronic health record system. A strong relationship between electronic health record system usability and the odds of physician burnout was also observed [16].

The Pragmatic Adaptive Trial for Respiratory Infection in Children (PATRIC) was established at a tertiary pediatric hospital to collect prospective data from both parents and health care providers and to assist in the understanding and management of pediatric ARI in the ED and in the community. The first step in PATRIC was the development of a patient registry (PATRIC Registry) that aimed to integrate digital tools into the workflows to enhance engagement with parents and clinicians. Sharing of study information, consent, and follow-up surveys were conducted using REDCap (Research Electronic Data Capture) tools [17,18]. REDCap is a secure, internationally used web-based platform designed to support data capture for research studies.

A second web-based system, Parent Engagement through Technology Solutions (PETS), was used allowing physicians to create personalized discharge instructions for 6 common pediatric ARI diagnoses. These personalized instructions were accessible to parents on a mobile phone or could be printed if an electronic option was not acceptable to the parent.

Before the launch of the PATRIC Registry, we conducted sessions with parents and physicians that comprised semistructured interviews as well as usability testing of our mobile and desktop interfaces.

The aims of this study are to understand parent and clinician experience of discharge communication and engagement in clinical research and to determine the efficacy of 2 different digital tools integrated into a pediatric patient registry for ARI.

## Methods

### Overview

This study was carried out over 2 weeks in September 2019 at Perth Children’s Hospital (PCH) and at the Telethon Kids Institute located in Perth, Western Australia. PCH is the sole tertiary pediatric hospital for the state of Western Australia. The PCH ED has approximately 70,000 visits per year. Research on patterns of presentations to 11 Australian and New Zealand pediatric EDs has demonstrated that the most common diagnoses are for infectious (usually viral) respiratory infections [19].

The Telethon Kids Institute is a child health research institute focused on the prevention and management of pediatric childhood diseases and is colocated with PCH.

The human research ethics committee of the Child and Adolescent Health Service approved this study (RGS3078). All participants received a participant information sheet and provided written consent to be part of the study.

### Study Design and Setting

#### Exploratory Study

The study was designed as an exploratory study. Each session with parents and physicians featured (1) a semistructured interview, (2) usability testing of system interfaces with direct observation, and (3) completion of the SUS.

#### Semistructured Interviews

All participants were interviewed with 2 research team members present. A researcher asked predetermined questions, and the other researcher acted as notetaker. RP, BB, and SD took turns as interviewer and notetaker.

#### Usability Testing

Usability testing is the direct observation of participants completing a series of tasks and measuring speed, accuracy, and understanding (as well as other specific, largely qualitative results). Usability testing methods are widely used in the design and development of digital interfaces, especially for websites and software. In formulating the script and materials for usability testing of the interfaces, the team relied on guidance from a user experience design consultant with field usability testing expertise (SH).

#### SUS Scores

On completion of direct observation, all participants rated the interface with the SUS, a widely used Likert-type 10-question survey measuring a user’s satisfaction where responses are converted into an overall usability score out of 100 [20]. SUS scores can be converted into equivalent school grades, with scores >90 representing a grade of *A* and a score of 0 to 60 representing a grade of *F* for usability [21,22].

### Digital Tools

We integrated 2 digital systems into the PATRIC Registry. The first was a web-based digital discharge communication system allowing physicians to choose from disease-specific templates using a desktop interface to create personalized discharge instructions that can be sent to a parent’s mobile phone. The system, first piloted in an adult ED in 2018 [23], is referred to as PETS for discharge communication. Content for 6 common diagnoses of ARI was developed for the PATRIC Registry with senior physician and consumer input. Discharge instructions were written at a level of readability appropriate for the general population, with accompanying pictograms targeted at those with low health literacy (Multimedia Appendix 1).

The second digital system used for the PATRIC Registry was the REDCap platform. PATRIC Registry parent information, including a 2-minute explainer video, electronic consent, and surveys, was developed for mobile phones using the existing functionality of REDCap (Multimedia Appendix 2). Digital copies of the PATRIC Registry information sheet and electronically signed consent form were automatically sent to the email address of the parent using the mobile interface.

### Participants

The ideal sample size for usability testing methods is highly debated. A literature review on this topic advises that a sample size range from 5 to 10 participants is likely to be effective for usability studies focused on problem discovery. Accordingly, we aimed at recruiting 8-10 participants for each group [24]. Participant demographic information was not collected, and all responses and comments were deidentified.

To recruit parent participants, an email invitation was sent to all current nonclinical staff members at the Telethon Kids Institute.

A convenient sample of staff members who were parents of children aged 1-12 years with no detailed knowledge of the technology workflows of the PATRIC Registry were enrolled in the study. A total of 12 parents expressed interest through email in participating in the study, and 11 parents were scheduled over 4 days for a 30-minute session that featured semistructured interviews followed by interface usability testing for both the PETS mobile interface and the REDCap mobile interface. One parent was unavailable during the allocated time slots scheduled for usability testing; therefore, they did not participate in the study.

The inclusion criteria for clinician participants were being a physician currently employed on rotation or as a permanent staff member in the ED at PCH with no prior knowledge of the technology workflows of the PATRIC Registry. A convenient sample was used whereby a senior ED physician and research team member (KB) asked physicians meeting the inclusion criteria whether they would be interested in participating in the study. A total of 8 physicians agreed to participate, and all sessions were carried out on the same day in a nonclinical area of the ED.

### Semistructured Interviews

Semistructured interview questions for parents were focused on experiences after visiting an ED with a child. If the participant had no experience of attending an ED with a child, they were asked to reflect on an experience after a family physician visit with a child. They were also asked about their experiences of information seeking and technology use after an ED visit. Further questions centered around the parent experience of information-seeking and engagement in clinical research studies (Multimedia Appendix 3).

Interview questions for physicians were based around their experience of discharge practice and different modes of communication at discharge (Multimedia Appendix 4).

The Framework Method [25] using Microsoft Excel was used to analyze participant responses from the semistructured interviews with parents and physicians. Authors SD, RP, and SJC met to discuss the development of a working analytical framework. Authors SD and RP then independently familiarized themselves with all response notes and coded the responses to identify important issues in the data set. Although areas of interest had been identified for the study, both authors sought to identify any unexpected perspectives within the response data. Both authors then charted the data into the Framework Method matrix and met again with author SJC to further discuss how the data had been charted and to identify the main themes of the data. This was followed by discussions about the main themes, impressions, and ideas based on the data, which led to a written analysis.

### Usability Testing

The usability testing component of the sessions followed the semistructured interview.

All participants were directly observed carrying out a series of predetermined tasks using the interface. The *think aloud* method was also used where participants were actively encouraged to “verbalize their thoughts while performing a computer-supported task” [26].

Each task consisted of any combination of one to three actions: *Start Action*, *Find Method*, and *Select Method*. *Start Action* represented the initiation of a task, such as pressing a button or opening a calendar picker. *Find Method* represented the way a user searched for information, such as using a QR code, browsing, or searching, and *Select Method* related to response actions such as use of a radio button or typing.

Task outcomes were then recorded as *success*, *failure*, *software failure*, or *not observed*. Tasks *not observed* usually described noncompulsory tasks; for example, where a participant was invited to add a comment. Notes on the outcomes of all tasks as well as successes and difficulties with task completion were recorded.

To test the PETS mobile interface, parents were supplied with a mobile phone similar to their personal mobile phone (Android or iOS) to protect privacy. They were then given a fictional scenario to read about a child presenting to the ED with community-acquired pneumonia. Parents were asked to assume that their treating ED physician had sent some discharge instructions for their child to their mobile phone. They were then asked to access the instructions on the mobile phone using a link embedded in an SMS text message.

For usability testing of the REDCap mobile interface, parents were given the same fictional scenario of a child presenting to the ED with community-acquired pneumonia and asked to assume that they were interested in finding out more about the PATRIC Registry. They were observed using the REDCap mobile interface to learn more about the PATRIC Registry and to electronically provide consent for their child to take part.

For usability testing of the PETS desktop interface, the ED physicians were given a fictional scenario of a child with mild community-acquired pneumonia mocked up on a routine preprinted ED triage sheet. Physicians were then asked to create discharge instructions using the PETS desktop interface on a desktop computer. Following creation of the instructions, the physicians were asked to send the instructions to the parent’s mobile phone.

### Mean Usability SUS Score

After being directly observed using their respective interfaces, all parent and physician participants completed the SUS. In this study, the participants’ individual SUS scores were added and then divided by the number of participants to give a mean usability SUS score for each interface tested.

## Results

### Semistructured Interviews

#### Experience of Discharge

Most parents (9/11, 82%) indicated that they receive verbal discharge information for their child when visiting an ED or a family physician. Some parents (5/11, 46%) mentioned having received preprinted discharge information in addition to verbal information about their child’s condition. Of the 11 parents, 3 (27%) described anxiety about the ability to recall all the information given verbally, particularly in regard to what to do if a child’s condition deteriorated after a visit.

When the parents were asked how they would seek further information after an ED or family physician visit with their child, many (8/11, 73%) mentioned using the internet or *Dr Google* (4/11, 36%). Many of the parents (8/11, 73%) stated a preference for receiving discharge information through digital technologies. An unexpected theme was the importance of being able to share information with a partner or other caregiver (3/11, 27%).

In describing their discharge practice, the physicians commonly mentioned giving verbal information (7/8, 88%). All physicians interviewed sometimes used accompanying printed information in the form of a template document or condition-specific health fact sheet either locally sourced or obtained from a reputable tertiary pediatric hospital website. Physicians mentioned a wide variety of challenges with the provision of discharge instructions, such as the amount of time required to provide comprehensive verbal instructions (3/8, 38%) and concerns around some parents’ understanding of written discharge materials (2/8, 25%). Of the 8 physicians, 1 (13%) highlighted a need for discharge materials in languages other than English.

#### Experience of Participation in Clinical Research

In regard to participation in clinical research studies, most of the parents (9/11, 82%) suggested various digital technologies as a preferred way for engaging with research initiatives. Some of the parents (5/11, 46%) specifically mentioned a website as the preferred technology for engagement. Some of the parents expressed frustration associated with participation in clinical research studies, including a lack of complete information (2/11, 18%) and too much information and difficulties contacting study staff (1/11, 9%). Several (4/11, 36%) of the parents wanted initial personal information from researchers or the physician. Many (7/11, 64%) of the parents mentioned the need for electronic surveys to be short and easy to fill out, and others (4/11, 36%) found it frustrating to be asked to enter free-text responses or comments.

### Usability Testing

#### PETS Mobile Interface for Parents

**Figure 1:**
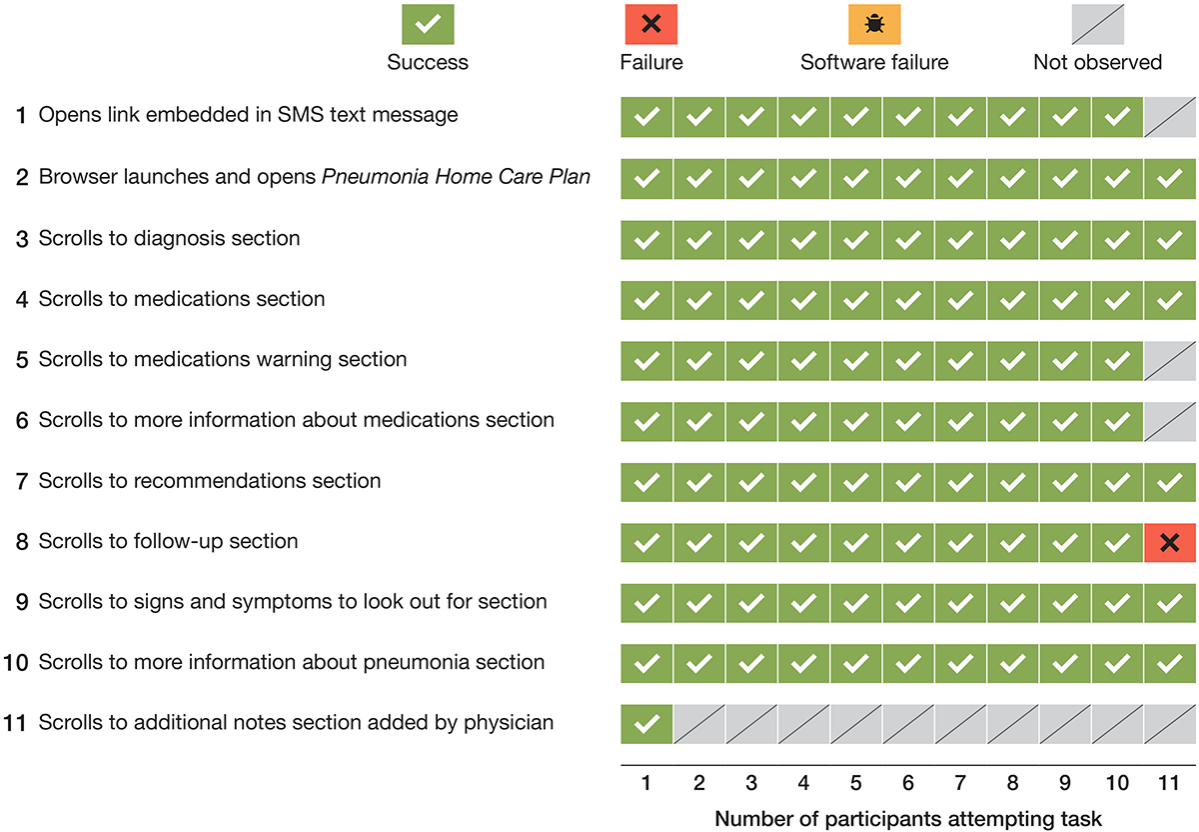
Task outcomes for parents using the PETS mobile interface for discharge instructions (N=11). PETS: Parent Engagement through Technology Solutions.

Each of the 11 parents was directly observed using the PETS mobile interface to complete 11 tasks. Of a total of 121 tasks undertaken by all parents, 107 (88.4%) were a *success*, 1 (0.8%) was a *failure*, no task was ascribed to *software failure*, and 13 (10.7%) were *not observed* (Figure 1).

#### PETS Desktop Interface for Physicians

**Figure 2:**
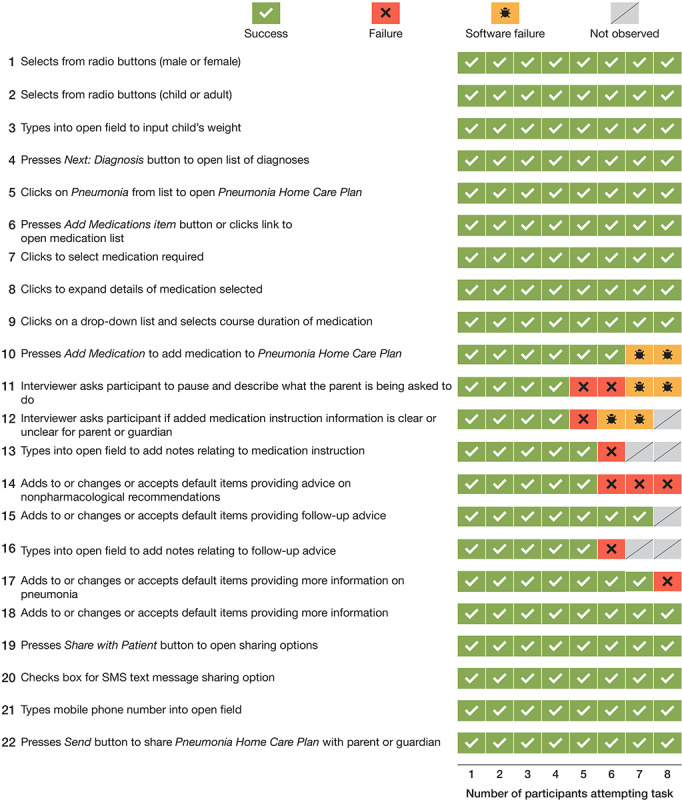
Task outcomes for emergency department physicians using the PETS desktop interface for creating discharge instructions (N=8). PETS: Parent Engagement through Technology Solutions.

A total of 8 ED physicians were observed carrying out a series of 22 tasks using the PETS desktop user interface. Of a total of 176 tasks, 155 (88.1%) were a *success*, 9 (5.1%) were a *failure*, 6 (3.4%) were ascribed to *software failure*, and 6 (3.4%) were *not observed* (Figure 2).

Of the 9 failures that were *not due to software*, the most common (3/9, 33%) occurred when physicians selected an add or edit option for the nonpharmacological advice section of the instructions. The drop-down list of options to choose from included several options not relevant for the diagnosis of pneumonia.

Other examples of failures *not due to software* occurred in relation to input into free-text fields. For example, of the 8 physicians, 1 (13%) was unsure of the amount of content typed into a free-text field because of a restriction of the field-viewing window.

In all, 2 *software failures* occurred when 1 of the 2 methods to add a medication to the instructions did not function because a button was inactive, whereas 4 *software failures* were due to the incorrect function of an external link, where a new tab did not appear in the browser window. This required the ED physicians to use the browser’s *back* button to preserve their work.

### REDCap Mobile Interface for Parents

**Figure 3:**
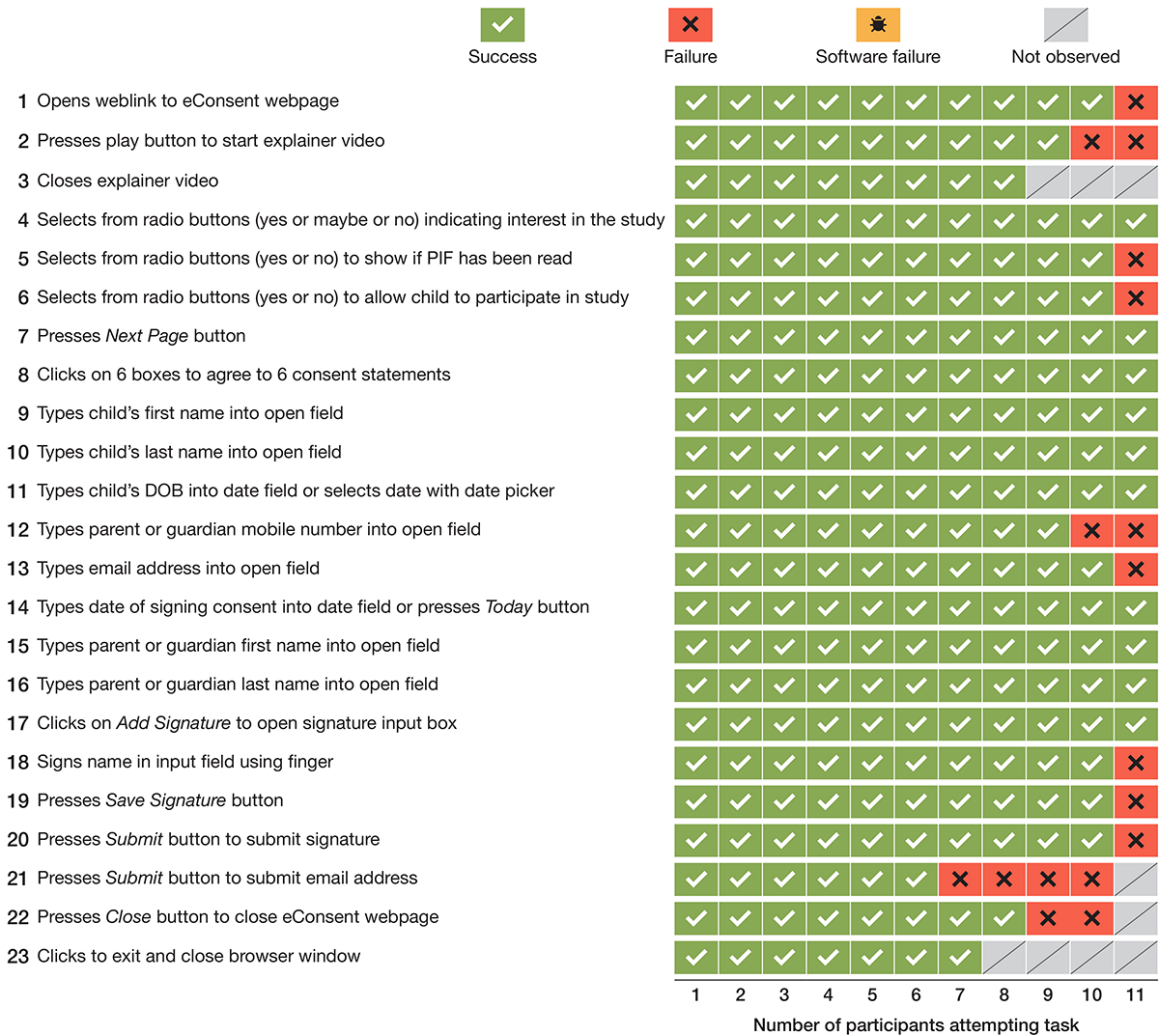
Task outcomes for parents using the REDCap (Research Electronic Database Capture) mobile interface for the eConsent process (N=11). PIF: participant information form; DOB: date of birth.

For the REDCap mobile interface, parents were directly observed attempting a series of 23 tasks that involved accessing PATRIC Registry information and completing the electronic consent process. Of a total of 253 tasks, 226 (89.3%) were a *success*, 18 (7.1%) were a *failure*, none were due to *software failure*, and 9 (3.6%) were *not observed* (Figure 3).

Several of the failures occurred when parents attempted to enter their mobile phone number in a format that did not conform to the required field format. Other failures occurred when parents had not noticed the link to the PATRIC Registry explainer video and had not entered email address details.

### SUS Mean Scores

**Figure 4:**
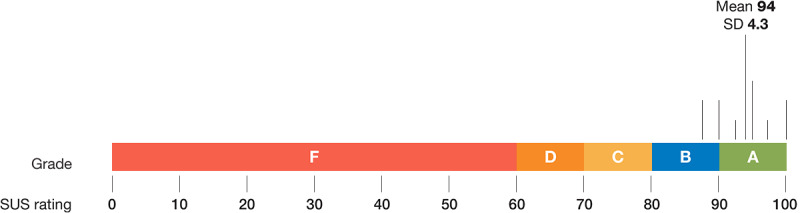
Mean SUS score and equivalent grade given to the PETS mobile interface by parents (N=11). SUS: System Usability Scale; PETS: Parent Engagement through Technology Solutions.

Parents gave the PETS mobile interface for discharge instructions a mean score of 94 out of 100 (SD 4.3) on the SUS, which is equivalent to an *A* grade (Figure 4).

**Figure 5:**
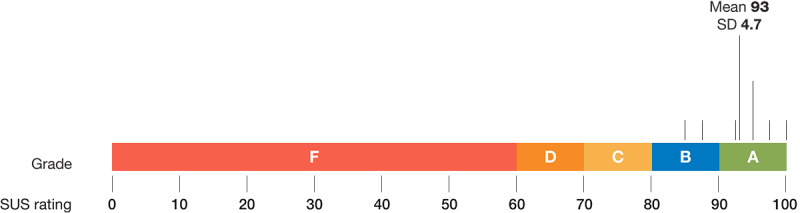
Mean SUS score and equivalent grade given to the PETS desktop interface by physicians (N=8). SUS: System Usability Scale; PETS: Parent Engagement through Technology Solutions.

ED physicians gave the PETS desktop interface a mean score of 93 out of 100 (SD 4.7) on the SUS, which is equivalent to an *A* grade (Figure 5).

**Figure 6:**
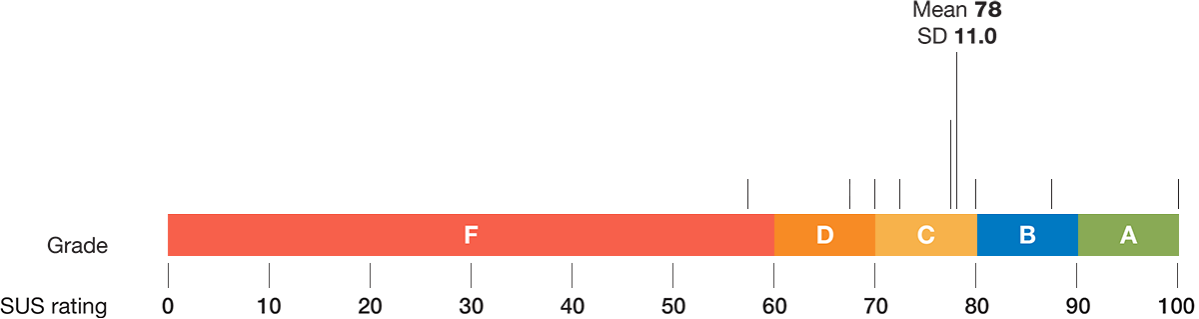
Mean SUS score and equivalent grade given to the REDCap (Research Electronic Database Capture) mobile interface by parents (N=11). SUS: System Usability Scale.

Parents gave the REDCap mobile interface a mean score of 78 out of 100 (SD 11.0) on the SUS, which is equivalent to a *C* grade for usability (Figure 6).

## Discussion

### Principal Findings

To our knowledge, this is the first study conducted to determine the efficacy of digital tools for discharge communication and clinical research engagement with parents and physicians before the launch of a pediatric patient registry for ARI. Analysis of semistructured interview responses using the Framework Method helped to identify communication challenges around discharge communication and clinical research engagement. Software interface usability testing methods and the use of the SUS helped to identify potential addressable issues for users of our digital tools before the launch of the patient registry.

In prior experience of the discharge process, parents expressed concern regarding their ability to correctly recall and follow all verbal discharge instructions given to them. This highlights a known need for improvement to current discharge communication practice, supported by recent evidence in a systematic review demonstrating that many parents make errors related to knowledge and execution of ED and inpatient discharge instructions [27].

Parents in our study also indicated a need for written advice to help reinforce verbal discharge advice. Current literature suggests that providing written instructions accompanied by verbal advice improves patient recall and comprehension [28-30], although this is not standard in current practice, as demonstrated in our study. Parents in our study highlighted technology as a viable option to reinforce verbal discharge advice, reflecting the widespread uptake of health technology in everyday life over the past 10 years [8,31]. The COVID-19 pandemic has further hastened the adoption of digital health tools. For example, telehealth visits for patients on Medicare in the United States increased from 13,000 weekly visits before the pandemic began to 1.7 million weekly visits in April 2020 [8]. Despite this unprecedented interest and uptake, the question is yet to be answered as to which form of the emerging digital tools is the most effective for improving discharge communication [32,33].

In our study, physicians described challenges associated with the time required to provide comprehensive verbal discharge instructions. Dean et al [34] suggest that although ED physicians are cognizant that effective communication with patients demands establishing rapport and ensuring comprehension, many physicians find themselves prioritizing efficiency in the time-pressured and chaotic environment of the ED. This is reflected in a study where verbal discharge communication in the ED was recorded and medical staff took an average of 76 seconds to impart verbal discharge advice to patients [35]. Digital tools may assist physicians to provide discharge information in a timely manner to patients. More research is required to determine how this might best be achieved.

Physicians raised concerns around the level of parent understanding of written discharge information. There is limited evidence demonstrating how often parents read written materials given to them. In one US study, only a few parents read through their child’s written discharge instructions and Hispanic families and those without health insurance were least likely to read the instructions [36]. Evidence also suggests many printed materials are written at levels of readability that are too high for the general population, making them inaccessible for those with low health literacy and limited English proficiency [37,38].

In exploring experience of engagement with clinical research studies, parents described the importance of having a reliable and central source of information. Similar to feedback on discharge advice, they mentioned electronic options such as a website, Twitter, or email as viable for information seeking. A 2021 US review of digital tool use in clinical research states that in the past 5 years “digital health technologies have exploded” and are now increasingly being integrated into clinical trials operations and that this accompanies the ubiquity of smartphone technology use [8].

The usability component of our sessions, featuring direct observation of participants using the software interfaces, was successful in identifying potential issues with our digital tools before the launch of the PATRIC Registry; for example, 1 of 2 methods allowing a physician to add a medication to the discharge instructions was nonfunctional (the button did not work). Interface usability testing allowed this to be easily identified so that it could be addressed before the launch. Furthermore, the *A* grades on the SUS for both the mobile and desktop PETS interfaces confirmed that parent and physician users endorsed the use of the PETS system for providing discharge instructions.

The REDCap system mobile interface for eConsent and survey completion was less favorably received by parents, who gave it a *C* grade. Task failures with inputting mobile number and email address details were deemed critical, given that this information is key to successful clinical trial enrollment and ongoing parent engagement. Best practice for web-based form design suggests that entry help by way of stating the rule imposed on a restricted field for a mobile phone number or an email address can lead to fewer input errors [39]. The failure of several parents to notice the link to the explainer video was also an important insight. Design recommendations suggest that users are more likely to see hyperlinks on an uncluttered layout with larger font sizes and simple terminology [40,41]. Although some issues concerning the usability of the REDCap mobile interface would not be easily solved because of the limitations around changing design features of an existing and widely used technology, we nevertheless found that usability testing was effective in identifying these and other addressable issues before the launch of the PATRIC Registry.

Unlike health care, industries such as aviation have made use of well-established computer science practices based on user-centered design theories that aim to create a positive experience for users of new digital tools [15,42]. The COVID-19 pandemic has led to a significant increase in the use of digital tools for clinical research purposes and for delivering clinical care [8]. This moment offers the health care industry a key opportunity to develop more efficient and usable digital tools for patients and clinicians.

Pediatric ARI places a huge burden on the community both in Australia and worldwide. Initiatives such as the PATRIC Registry rely on the engagement of parents and physicians to help better understand and manage ARI in the community.

This study assisted us to better understand parent and physician experiences around discharge communication and participation in clinical research. The study also serves as an example of how clinical researchers can adopt an interdisciplinary approach with user experience experts to integrate qualitative research methods and interface usability testing methods in determining the efficacy of digital tools in the pediatric clinical research setting.

### Limitations

We decided to have a notetaker to create a record of participants’ comments and feedback rather than creating a video recording of each session for posttesting transcription. Note-taking may have introduced bias because we relied on the notetaker’s records rather than on a direct transcription of each participant’s comments for our analysis. Similarly, we chose not to adopt screen-recording software to measure the time taken by users to complete tasks, meaning that we were unable to include this metric.

In terms of selection of participants, parent participants came from a convenient sample of staff working at a colocated research institute. Parents who are nonclinical research staff may have higher levels of education and health literacy and be more familiar with clinical research processes than parents selected from the general population. The parent group who volunteered their time for the study may have also had significantly more interest in the use of technology in clinical research than other institute staff members.

Not all parents had experienced a visit to an ED with their child. If this was the case, we asked parents to reflect on experiences visiting their family physician with their child. It is possible that the family physician experiences differed significantly from ED experiences. Similarly, feedback from a parent with lived experience of a child with pneumonia as presented in our case study may have differed from a parent who had not experienced this.

Physician participants also came from a convenient sample. This may have resulted in a group more open to the use of new technology and discharge communication than a randomly selected sample group of physicians from the ED.

Finally, our tools are currently only in English, and our assessment was limited to participants who could read, speak, and write English. Further work in this area is important to understand and cater to the needs of those who access pediatric EDs with limited English proficiency in terms of discharge communication and participation in clinical research.

### Conclusions

This study shows the feasibility of combining qualitative research methods with software industry interface usability testing methods to help determine the efficacy of digital tools in a pediatric clinical research setting.

Analysis of semistructured interview responses using the Framework Method allowed us to better understand parent and physician experiences of discharge communication and clinical research engagement. Technology was identified by parents as a viable means to reinforce discharge advice and for engagement in clinical research. Software interface usability testing methods and the use of the SUS assisted us in gauging the efficacy of our digital tools with parent and physician users before the launch of our pediatric registry.

## Appendix

Multimedia Appendix 1 Example of pneumonia discharge instructions on the Parent Engagement through Technology Solutions mobile interface for parents.

Multimedia Appendix 2 Example of eConsent on the REDCap (Research Electronic Database Capture) mobile interface for parents.

Multimedia Appendix 3 Semistructured interview questions for parents relating to discharge and clinical research experience.

Multimedia Appendix 4 Semistructured interview questions for physicians relating to discharge.

## Abbreviations

ARI: acute respiratory infection
ED: emergency department
PATRIC: Pragmatic Adaptive Trial for Respiratory Infection in ChildrenPCH: Perth Children’s Hospital
PETS: Parent Engagement through Technology Solutions
REDCap: Research Electronic Database Capture
SUS: System Usability Scale
