# Supplementary material for: Efficacy of Digital Health Tools for a Pediatric Patient Registry: Semistructured Interviews and Interface Usability Testing With Parents and Clinicians
Source: JMIR Form Res. 2022 Jan 17;6(1):e29889. doi: 10.2196/29889 (PMC8804961; doi:10.2196/29889)

Multimedia Appendix 2: Example of eConsent on the REDCap (Research Electronic Database Capture) mobile interface for parents

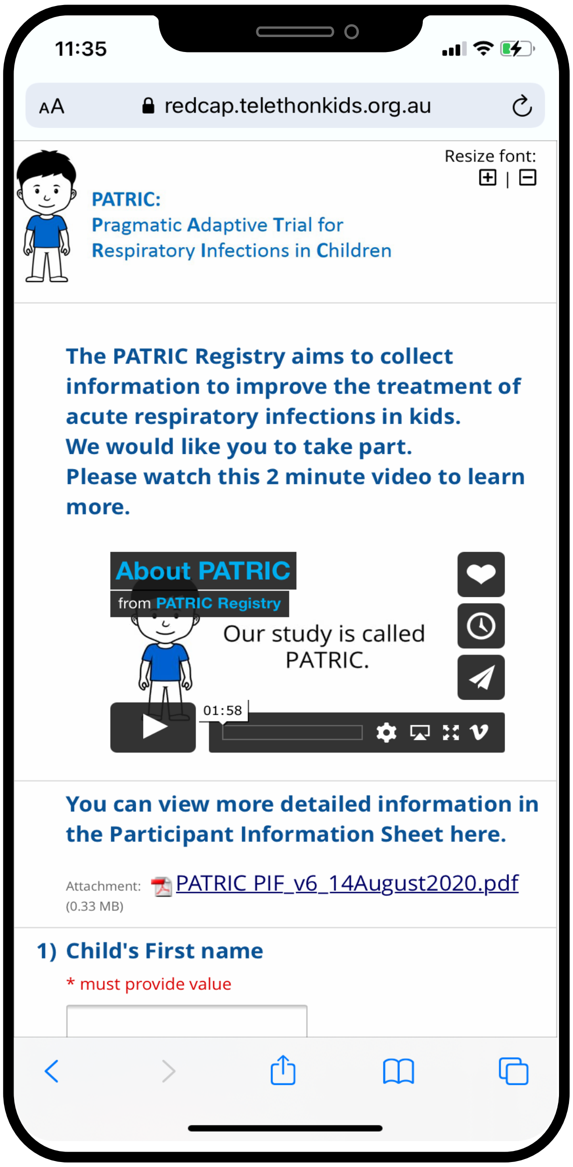

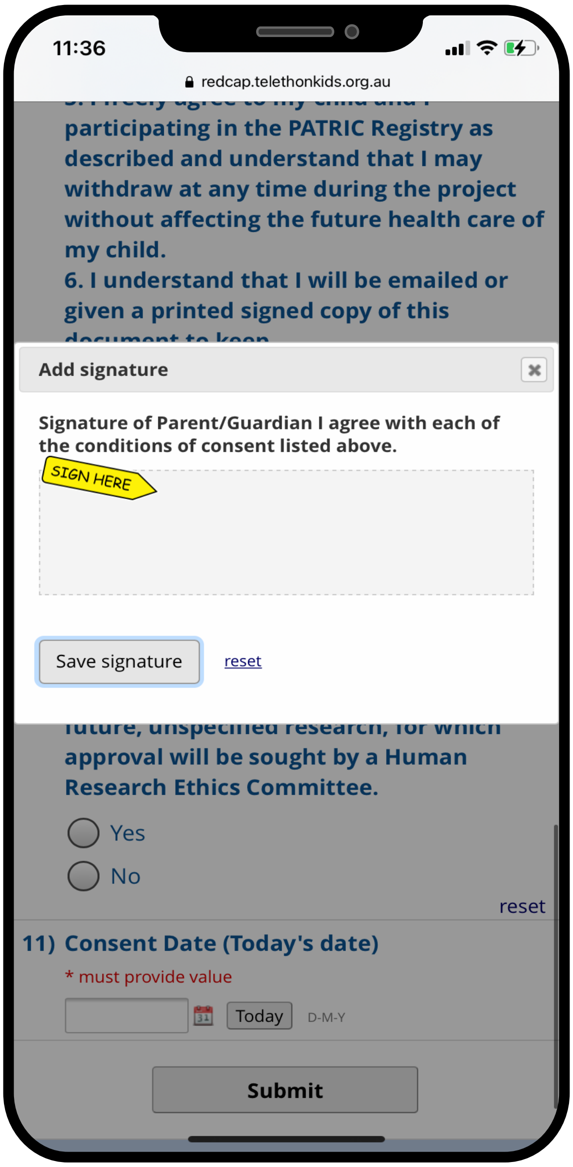

Supplement: Multimedia Appendix 2 [file formative_v6i1e29889_app2.pdf]
