# Supplementary material for: Efficacy of Digital Health Tools for a Pediatric Patient Registry: Semistructured Interviews and Interface Usability Testing With Parents and Clinicians
Source: JMIR Form Res. 2022 Jan 17;6(1):e29889. doi: 10.2196/29889 (PMC8804961; doi:10.2196/29889)
Supplement: Multimedia Appendix 3 [file formative_v6i1e29889_app3.pdf]

Multimedia Appendix 3: Semistructured interview questions for parents relating to discharge and clinical research engagement

Semi-structured questions for parents relating to discharge experience

1. How do you get answers to questions about care for an illness about your child after you get home from a doctor visit?
2. Do you own a mobile phone?
3. What is the best description of how frequently you use a mobile phone?
4. Do you have a computer at home?
5. Do you have a printer at home?
6. Which is the best description of how frequently you use a personal computer?
7. What is the most effective way for you to get home care information for your child after a doctor visit?
8. What is the most frustrating thing about getting home care information after a doctor visit right now?

Semi-structured questions for parents relating to experience of participation in clinical research studies

1. How do you prefer to find out information about a research study that you are considering enrolling in?
2. What is the most frustrating thing about getting information about a research study?
3. Have you experienced filling out surveys on your mobile phone?
4. What is the most frustrating thing about filling out surveys on your mobile phone?
