# Supplementary material for: Efficacy of Digital Health Tools for a Pediatric Patient Registry: Semistructured Interviews and Interface Usability Testing With Parents and Clinicians
Source: JMIR Form Res. 2022 Jan 17;6(1):e29889. doi: 10.2196/29889 (PMC8804961; doi:10.2196/29889)
Supplement: Multimedia Appendix 4 [file formative_v6i1e29889_app4.pdf]

Multimedia Appendix 4: Semistructured interview questions for physicians relating to discharge

1. How do you usually provide discharge instructions to parents and carers leaving the ED?
2. What is the most frustrating thing about providing discharge instructions to parents and carers?
